# Understanding Wine through Yeast Interactions

**DOI:** 10.3390/microorganisms9081620

**Published:** 2021-07-29

**Authors:** Evangelia A. Zilelidou, Aspasia Nisiotou

**Affiliations:** Institute of Technology of Agricultural Products, Hellenic Agricultural Organization “Demeter”, Sofokli Venizelou 1, GR-14123 Lykovryssi, Athens, Greece; liazilelidou@gmail.com

**Keywords:** alcoholic fermentation, wine yeasts, *Saccharomyces cerevisiae*, non-*Saccharomyces* yeasts, yeast competition, biotic stress, yeast co-culture

## Abstract

Wine is a product of microbial activities and microbe–microbe interactions. Yeasts are the principal microorganisms responsible for the evolution and fulfillment of alcoholic fermentation. Several species and strains coexist and interact with their environment and with each other during the fermentation course. Yeast–yeast interactions occur even from the early stages of fermentation, determining yeast community structure and dynamics during the process. Different types of microbial interactions (e.g., mutualism and commensalism or competition and amensalism) may exert positive or negative effects, respectively, on yeast populations. Interactions are intimately linked to yeast metabolic activities that influence the wine analytical profile and shape the wine character. In this context, much attention has been given during the last years to the interactions between *Saccharomyces cerevisiae* (SC) and non-*Saccharomyces* (NS) yeast species with respect to their metabolic contribution to wine quality. Yet, there is still a significant lack of knowledge on the interaction mechanisms modulating yeast behavior during mixed culture fermentation, while much less is known about the interactions between the various NS species or between SC and *Saccharomyces* non-*cerevisiae* (SNC) yeasts. There is still much to learn about their metabolic footprints and the genetic mechanisms that alter yeast community equilibrium in favor of one species or another. Gaining deeper insights on yeast interactions in the grape–wine ecosystem sets the grounds for understanding the rules underlying the function of the wine microbial system and provides means to better control and improve oenological practices.

## 1. Introduction

**Figure 1:**
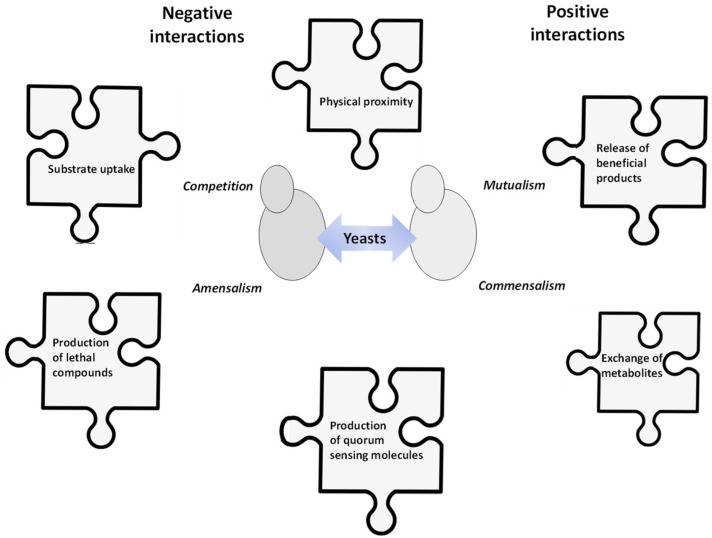
The puzzle of wine yeast interactions.

Every niche on earth, from the deep layers of soil and oceans to the upper atmosphere, is inhabited by microorganisms that rarely function as single entities. The study of microbial interactions in food products and wine is of particular interest. Wine constitutes an excellent model to examine the effect of microbial associations on the formation of the organoleptic characteristics of a fermented product. Due to grape must richness in microbial species along with its particular physicochemical properties subjected to highly dynamic microbial-driven alterations, wine is undoubtedly a product of microbial interactions. During the initial stages of alcoholic fermentation, several yeast species and strains are present in the must [1]. Species abundance and diversity are decisive in the formation of wine sensorial attributes. The grape-originating yeast community is further subjected to modifications during fermentation. Inter-species and -strain fitness differences towards environmental factors as well as their interactions define the spatiotemporal occupation of the substrate [2,3]. Yeast–yeast interactions may be just as important as the composition of the grape yeast consortium, influencing the population variation and final dominance in wine. Different types of yeast interactions (e.g., mutualism and commensalism or competition and amensalism) may exert positive or negative effects, respectively, on the populations [4] (Figure 1; Table 1). Positive interactions may be mediated via the modification of the growth environment through metabolite exchange (e.g., acetaldehyde overproduction by one species may be utilized by another yeast via redox interactions and regeneration of NAD^+^ from NADH and acetaldehyde) [5]. Release and accumulation of certain compounds (e.g., amino acids) by one species may also prove beneficial for other species [4]. On the other hand, the faster and more efficient use of nutrients (nitrogen, glucose, vitamins, metal ions) or oxygen intake by particular yeasts may negatively influence the fitness of species with weaker substrate uptake capacity [6,7]. Release of antimicrobial compounds such as killer toxins, acetic acid, ethanol, short-chain fatty acids or low-mass peptides may as well contribute to the growth arrest or even death of yeasts during wine fermentation [8,9,10]. Physical contact of yeast cells appears to be another mode of interaction via which certain yeast species may regulate the presence and the persistence of other species [11,12]. The exact mechanisms behind contact-mediated interactions have not been elucidated yet. Cellwall-related modifications have been proposed to modulate cell–cell contact. Specifically, the expression of cell–cell adhesion-associated proteins encoded by the *FLO* gene family has been linked to contact-dependent yeast interactions in multispecies yeast ecosystems [13]. Finally, specific molecules such as aromatic alcohols (e.g., tryptophol) have been hypothesized to have quorum-sensing properties and thus the potential to control yeast populations [14,15]. Quorum sensing as a cell signaling-based mode of interaction supports the concept of cell communication via the production of specific compounds, which above a threshold regulate population behavior. Although this mechanism has often been proposed as being involved in wine yeast interactions, its role still remains debatable [16].

The acknowledgement of the importance of yeast interactions, in combination with the reevaluation of the role of non-*Saccharomyces* (NS) yeasts in the formation of wine identity, has challenged the wine microbiologists to look into the function of interacting yeast communities. Currently, the line in research is drawn by the need to manage certain oenological aspects (e.g., the acetic acid or ethanol levels, the total acidity, etc.) and to achieve distinct sensory profiles of the wine without compromising the completion of fermentation [8,17,18]. However, despite the fact that significant progress has been made towards the collection of information on practical aspects of co-cultivating NS with *Saccharomyces cerevisiae* (Sc) yeasts during wine making, the role of NS–NS interactions has been overlooked. Most importantly, the literature still lacks information regarding the respective molecular mechanisms involved. The objective of this review is to summarize the recent scientific knowledge on yeast–yeast interactions during wine making and to highlight their impact on the wine character. A brief outline of NS–SC interactions during mixed fermentations provides the latest documentations on this branch of yeast–yeast interactions. *Saccharomyces* interspecies interactions will be reviewed. The unexplored area of NS yeast interactions will be put forward. Special focus has been given on the mechanisms playing key roles in the performance of wine yeast starters in co-inoculation schemes.

**Table 1:** Type and mode of inter- and intra-species yeast interactions in wine.

Type of Interaction	Mode of Interaction	Yeast Species Association ^1^	Description of Interaction	Reference
Positive interactions (mutualism/commensalism)	Modification of environment via metabolite exchange (e.g., redox balance via acetaldehyde production)	Sc–Mp	Reorientation of carbon fluxes and modification of NAD^+^/NADH balance by Mp	[7]
		Sc–Sc × Su	Acetaldehyde production by Sc × Su and utilization by Sc	[5]
	Release of beneficial products (e.g., amino acids)	Sc–Td	Cys-3SH ^2^ release by Td and uptake by Sc	
				[19]
	Cell contact-dependent effects	Sc–Td	Sc growth stimulation in contact with Td	[20]
	Production of putative quorum sensing molecules (e.g., aromatic alcohols)	Sc–Sb	Changes in species associations dependent on the concentration of tryptophol and melatonin in the growth medium	[14]
		Sc–Td		[14]
	Unknown	Sc–Hv	Increased survival rate of Hv in the presence of Sc	[21]
Negative interactions (competition/amensalism)	Substrate uptake (e.g., nitrogen, glucose, oxygen)	Lt–Hu	Space occupation by Lt	[22]
		Sc–Lt	Possible faster nutrient uptake by Sc	[8]
		Sc–Hv	Oxygen uptake by Hv	[23]
		Sc–Td	Oxygen uptake and biomass production by Td	[24]
		Sc–Mp	Iron sequestration by Mp	[9]
		Sc–Su	Faster substrate uptake and higher growth rates of Su under low temperature	[25]
	Production of lethal compounds (e.g., killer toxins, short-chain fatty acids, peptides)	Ci–Pg	Peptides produced by Ci against Pg	[26]
		Ci–Db	Peptides produced by Ci against Db	[26]
		Sc–Hu	Peptides produced by Sc against Hu	[27]
		Sc–Td	Peptides produced by Sc against Td	[27]
		Sc–Su	Putative killer toxins produced by Sc	[28]
		Sc–Hu	Killer toxins produced by Sc	[29]
	Cell contact-dependent effects	Sc–Lt	Viability loss of Lt in contact with Sc	[11]
		Sc–Hu	Viability loss of Hu in contact with Sc	[30]
		Sc–Sk	Suppression of Sk in contact with Sc	[31]
		Sc–Sc	Contact-mediated Sc inter-strain dominance	[32,33]
	Production of putative quorum-sensing molecules (e.g., aromatic alcohols)	Sc–Sb	Changes in species associations dependent on the concentration of tryptophol and melatonin in the growth medium	[14]
		Sc–Td		[14]

^1^ Sc, *Saccharomyces cerevisiae*; Hv, *Hanseniaspora uvarum*; Mp, *Metschnikowia pulcherrima*; Td, *Torulaspora delbrueckii*; Sb, Starmerella bacillaris; Lt, *Lachancea thermotolerans*; Su, *Saccharomyces* uvarum; Ci, Candida intermedia; Pg, *Pichia guilliermondii*; Db, Dekkera bruxellensis; Sk, *Saccharomyces kudriavzevii*; Sc × Su, *Saccharomyces cerevisiae* × *Saccharomyces* uvarum natural hybrid. ^2^ Cys-3SH, cysteinylated conjugate precursor of 3-sulfanylhexan-1-ol.

## 2. NS–NS Interactions

Spontaneous alcoholic fermentation is driven by a diversity of indigenous NS yeast species, which compete or cooperate with each other until they are progressively replaced by *S. cerevisiae*, the species with the strongest fermentative capacity. The presence and succession of these species during the winemaking process is linked to the analytical profile of wine. Despite the fact that for a long time the NS yeasts present in the freshly crushed grape must have been considered as spoilage factors, their role has been reevaluated and constant efforts are being made for their exploitation in winemaking [34,35].

The contribution of each NS population to the total metabolic profile is not predetermined based on its particular physiological properties, but is a result of yeast associations during winemaking. Hence, the manner via which these yeasts interact with each other defines the composition of the species consortium at the different stages of winemaking and the organoleptic characteristics of the final product. Nevertheless, NS interactions have been overlooked, escalating the difficulties in understanding and predicting the effect of the wine microbiome in the fermentation process [21].

Pena et al. [26,36] found that low-mass peptides produced by *Candida intermedia* LAMAP1790 may have a selective antimicrobial effect against spoilage yeasts such as *Dekkera bruxellensis* and *Pichia guilliermondii*. The influence of *Metschnikowia pulcherrima* on the metabolism of other NS yeasts has pointed out its potential to exert an antagonistic effect against important yeast genera in winemaking, such as *Hanseniaspora* and *Pichia* [37]. The reasoning and hypotheses behind the antimicrobial activity of *M. pulcherrima* (i.e., production of pulcherrimin, an iron-chelating agent) have been recently reviewed [38]. Johnson et al. [22] investigated the effect of selected commercial strains of *Metschnikowia* spp., *Lachancea thermotolerans* and *Torulaspora delbrueckii* against *H. uvarum* during pre-fermentation cold soaking, a process used for the treatment of red grapes before alcoholic fermentation. Their results reveal a significant decrease in acetic acid production by *H. uvarum* in mixed cultures compared to the monoculture model, possibly related to growth inhibition of *H. uvarum* in the presence of competing species. Bagheri et al. [39] constructed a seven-species yeast consortium approximating a grape must ecosystem to assess the impact of each individual NS yeast on the course of fermentation, the yeast population dynamics and the profile of volatiles in synthetic grape juice. Their results suggest that the chemical profile of wines may be indeed influenced by complex indirect or direct interactions among different NS yeast species. They highlighted the role of cell density in shaping yeast community structure and hence in the formation of different aroma signatures in the wine. It has been demonstrated that the concentration of carbon, nitrogen or aromatic amino acids in synthetic must is related with the production of higher alcohols by NS wine yeast species such as *T. delbrueckii*, *M. pulcherrima* and *Starmellera bacillaris* [15]. Previous research revealed that these alcohols may act as quorum-sensing signaling molecules and growth-regulating factors [15], making their study in respect to NS yeast interactions important.

Despite recent improvements, our understanding of the effect of NS–NS interactions on the organoleptic profile of wine remains limited, particularly regarding the underlying mechanisms. For instance, the metabolic adjustment of several important wine species, such as *Hanseniaspora* spp. or *Starmerella bacillaris* during mixed-inoculated fermentations has been neglected. Furthermore, it would be of great importance to study possible intra-species strain–strain interactions, as different strains of the same species have been shown to co-evolve during wine fermentation.

## 3. NS–Sc Interactions

As aforementioned, the research regarding NS–Sc interactions is still mostly a practical approach of mixed-species inoculum strategies focusing on wine quality improvement. Detailed investigation on the role of competitive or even cooperative interactions on the outcome of mixed NS-Sc fermentations is less common. Therefore, the NS species which have been mainly tested are the ones well-represented in grapes and musts at different stages of fermentation. Among these yeasts *Hanseniaspora*, *Torulaspora*, *Lachancea* and *Metschnikowia* species have most frequently been chosen to participate in studies evaluating NS–Sc co-cultures [8,17,18,40].

### 3.1. Hanseniaspora *spp*.

Notorious for increasing the volatile acidity of the wine [41], yet well known for their potential to raise the levels of acetate esters [42], various species of the *Hanseniaspora* genus have been used in mixed fermentations with *Saccharomyces cerevisiae*. The presence and the high efficiency of enzymes, such as β-d-glucosidase, in *Hanseniaspora* spp. compared to other yeasts add to the aromatic character of wines and improve the output of alcoholic fermentation during early stages [40].

Several authors have recorded significant increases in the concentration of important aroma compounds, as well as improvement in wine color in fermentations with *H. vineae* or *H. uvarum* and *S. cerevisiae* [43,44]. A decrease in ethanol content in the presence of *Hanseniaspora* spp. was observed, which was not correlated with an increase in glycerol or acetic acid concentration [45,46]. The authors hypothesized that the sugars were consumed via the oxidative pathway for the production of other products or biomass.

Aeration may serve as a practice to enhance *Hanseniaspora* biomass production in mixed *Hanseniaspora*–Sc cultures. Yan et al. [23] studied the role of oxygen supplementation during the fermentation of French Colombard wine, highlighting the positive impact of oxygen on the yeast metabolism and on the formation of aroma compounds. Excessive oxygenation however may tip the balance against *S. cerevisiae* and lead to stuck fermentations. Therefore, a fine tuning is required, setting an oxygen threshold above which *Hanseniaspora* biomass production may be unnecessary or even undesirable. Correspondingly, Li et al. [29] pointed out the importance of choosing right when it comes to the selection of the *S. cerevisiae* strain and inoculation strategy for *Hanseniaspora*–Sc mixed fermentations. In that work, a simultaneous *H. uvarum*–Sc inoculation scheme involving killer *S. cerevisiae* strains was very effective for the elevated production of fruity esters in the final product, providing better control of the NS competing population. While high cell density and prolonged survival of *Hanseniaspora* are necessary for sufficient production of aroma elements, an overproduction of such compounds (such as ethyl acetate) may sometimes be unwelcome in the final product. An optimum balance regarding the proportion of inoculated populations is required; this balance is affected by nutrient uptake and may potentially lead to cooperative activities. Harlé et al. [47] observed a synergistic effect on the production of glycerol in mixed-culture fermentations of *S. cerevisiae* with *H. uvarum* or *H. opuntiae*, pointing out the improvement in the fermentation yield when biodiversity increases (overyielding hypothesis). On the other hand, a negative influence of *H. uvarum* viability was ascribed to physical proximity with *S. cerevisiae* [30]. The authors constructed a fermentor with two compartments, each filled with the culture of a single yeast species [48]. These two chambers were separated by a membrane that allowed metabolite exchange but not cell contact. Interestingly, the analytical profile (e.g., content of specific esters, ethyl acetate, n-propanol, higher alcohols) of the wine produced when the two species were physically separated was different compared to that produced by cells in contact.

Despite the fact that *Hanseniaspora* spp. are principal yeast species at the initial stages of fermentation, information regarding their metabolism and how it is modified to adjust to biotic pressure is still missing. Studies on the management of nutrient resources and the modulation of the *Hanseniaspora* transcriptome and metabolome in the presence of Sc and/or other NS species will contribute to untangling the complex yeast networks.

### 3.2. Torulaspora delbrueckii

The high fermentative potential of *Torulaspora delbrueckii* (Td) among NS yeasts has rendered it one of the most popular species when mixed fermentations with *S. cerevisiae* are considered. In co-inoculation with *S. cerevisiae*, it has been found to intensify wine aroma and improve its overall qualitative attributes [49]. One of its main biological weapons to withstand competition with *S. cerevisiae* is its high ethanol tolerance and, as stated recently, the strain-depending capacity to produce killer toxins [50,51,52]. Zhang et al. [53] compared the analytical profile of Cabernet Sauvignon red wines fermented by different (commercial or indigenous) *T. delbrueckii* strains in mixture with a *S. cerevisiae* strain. Regardless of the *T. delbrueckii* strain used, the presence of both populations increased the duration of fermentation. The dependence of a mixed fermentation performance on the NS strain used was highlighted in this study, which supported the exploitation of selected indigenous NS strains. Such strains may indeed be considered as strong competitors against *S. cerevisiae*, being well adapted to cope with the specific must environment.

On the other hand, the selection of *S. cerevisiae* strain may well be of equal importance. Zhang et al. [52] suggested that the associations between Td and Sc in a mixed system are linked to the characteristics of the *S. cerevisiae* strain that determine processes such as the formation of higher alcohols or acetate esters. Indeed, strain identity may be particularly significant for NS–Sc mixed fermentations due to the direct contribution of each strain to the entire metabolome. However, indirect effects related to the mode and the extent to which a strain interacts with other species may also introduce modifications in the wine chemical composition. In line with this, Ruiz et al. [54] concluded that during wine fermentation *T. delbrueckii* may influence the response of *S. cerevisiae* to nitrogen availability, thereby affecting the course of the fermentation process. Regarding access to nitrogen, Álvarez-Fernández et al. [55] suggested that *T. delbrueckii* initiates nitrogen utilization by activating metabolic pathways other than those preferred by *S. cerevisiae*, resulting in the consumption of the available nitrogen sources in a different order (e.g., first ammonium sulphate and then amino acids) compared to its competitor. This supports the co-existence and the prolonged activity of both populations in mixed fermentations. Previous findings [19] revealed that under sequential inoculation of *T. delbrueckii* and *S. cerevisiae* in Sauvignon Blanc must, both species retained viability during a prolonged period of the fermentation course. This, accompanied by an increased *T. delbrueckii* biomass production, led to co-operative interactions between the two species for the production of 3-sulfanylhexan-1-ol and its acetate 3-sulfanylhexyl volatile thiols, thereby increasing their concentration in wine.

In the context of yeast interactions, it would be of great interest to investigate the killer activity of *T. delbrueckii* strains and its contribution to their competitive fitness during alcoholic fermentations. The number of studies exploring the aromatic amino acid kinetics, the effect of nitrogen addition and the production of important metabolic compounds in mixed fermentations of *T. delbrueckii* and *S. cerevisiae* is limited. Given that wine composition is the consequence of intricate yeast interactions that cause the presence or absence of particular compounds, rather than just the addition of metabolites from each population individually, further research is needed to understand the preference of specific metabolic pathways by *T. delbrueckii* in the presence of *S. cerevisiae*. 

### 3.3. Lachancea thermotolerans

Due to its outstanding ability to produce lactic acid, *Lachancea thermotolerans* (Lt) has been applied for the acidification of wines and subsequently in the improvement of color intensity of red wine [8,56,57]. Additionally, Lt may also produce other metabolites or induce compositional alterations associated with a desired sensory profile in wines [18,58]. Therefore, among NS yeasts, Lt is used as a prominent entrant in co-inoculation modalities with Sc. Inoculated either simultaneously or sequentially with Sc, Lt has been shown to improve the structure and the volatile profile of various wines. In a recent study, Lt could, by an unknown to the authors mechanism, limit the fermentative production of the non-desirable vinylphenols by Sc in aged wines, thereby reducing the content of these compounds in wines sequentially inoculated with the two species [59]. In a study comparing the mixed cultures of different species (i.e., *M. pulcherimma*, *Pichia kluyveri* and *T. delbrueckii*) with Sc, the co-culture of Lt with Sc was superior in increasing the lactic acid, glycerol and ethyl ester concentrations while producing the lowest levels of ethyl acetate and reducing the ethanol content of wines. The reduction in ethanol content may indeed be another attribute of wines produced via Lt–Sc co-fermentations. Sgouros et al. [8] recorded a 1.6% ethanol reduction in Vilana wine following the use of a highly lactate-producing Lt strain in sequential inoculation with Sc. In the same study the highest levels of lactic acid ever noted in mixed sequential NS–Sc fermentations were reported. On the contrary, the contribution of Lt in the final product was restrained in a simultaneous inoculation scheme due to a disadvantage of Lt in competition with Sc.

Cell contact has been suggested and investigated as a key factor related to Lt–Sc competition. Petitgonnet et al. [12] compared the exo-metabolomes of single and mixed Lt–Sc cultures either in contact or physically separated by a membrane. It was shown that ester and fatty acid content were higher in the absence of contact between yeasts of the two populations and generally a distinct metabolic profile was generated depending on the type of the culture. Last but not least, the availability of nutrients is a driving force in microbial interactions. In line with this, the nitrogen uptake, in terms of preferred N sources and rate of N metabolism, by *S. cerevisiae* and *L. thermotolerans* obviously impacts the outcome of Lt–Sc competition. Hence, the behavior of *S. cerevisiae* following the inoculation of *L. thermotolerans* in grape must will be determined by the access to nitrogen sources. The availability and the type of these sources depend on their previous utilization by *L. thermotolerans*, thus impacting the performance of *S. cerevisiae* fermentation. On the other hand, under simultaneous inoculation schemes, *S. cerevisiae* may lead via nitrogen consumption to modifications in *L. thermotolerans* metabolism, or faster *L. thermotolerans* inactivation and drive-specific *L. thermotolerans*-associated metabolites in wine [60,61].

Though limited, there are reports regarding killer activity of *L. thermotolerans* strains [56]. This strain-specific trait, in combination with high lactic acid productivity, also a strain-dependent characteristic of the species, may potentially serve as a competitive asset for *L. thermotolerans*. The antimicrobial activity of lactic acid and the early acidification of grape must might offer a competitive advantage for the relatively long survival of *L. thermotolerans* during the fermentation course. However, the genetic mechanisms behind the high lactic acid production rates of *L. thermotolerans* remain unclear. Only recently, Sgouros et al. [8], by studying the *L. thermotolerans* lactate dehydrogenase genes (*LDHs*), showed an implication of *LDH2*, but not of other *LDHs* or alcohol dehydrogenase genes, in increased lactate production at the expense of ethanol.

### 3.4. Metschnikowia pulcherrima

*Metschnikowia pulcherrima* (Mp) is among the NS yeast species that could potentially be a protagonist in the yeast war battleground. A claim for antimicrobial activity of *M. pulcherrima* is based on its potential to release the antimicrobial substance pulcherriminic acid, which sequesters ferric iron and forms the red-maroon pigment pulcherrimin. Hence, *M. pulcherrima* reduces the iron bioavailability for its competitors. In addition, *M. pulcherrima* may exert immunity to Sc killer toxins [9,38,62]. Together with *T. delbrueckii*, it has been shown to produce Beta-lyase, which is responsible for thiol production. There is evidence for reduced acetate levels [7,63,64] in contrast to increased glycerol levels in wines fermented with *M. pulcherrima* along with *S. cerevisiae*. In fact, the increased glycerol content in mixed Mp–Sc fermentations has been demonstrated to be not only due to the *M. pulcherrima* activity but also due to its elevated production by Sc [7]. Earlier studies have ascribed the enhanced production of glycerol in the presence of *M. pulcherrima* to the induction of the glycerol-3-phosphate dehydrogenase 1 gene in Sc [65].

There are also recent reports suggesting a reduction in ethanol levels during mixed Mp–Sc fermentations, an observation potentially attributed to the diversion of sugar conversion from ethanol towards the formation of other products. The accumulation of specific metabolites (e.g., fumarate) in monocultures of *M. pulcherrima* and their respective depletion in mixed Mp–Sc fermentations led to the conclusion that metabolite exchange between the two populations takes place. Interestingly, the combined use of these two species could modify the way by which nutrient availability affects the formation of metabolic compounds. While the production of specific compounds is only related to the potential of each species to produce them, many of the observed metabolomic differences in the media fermented with pure vs. mixed Mp–Sc cultures may be associated with species interactions and the manner in which these interactions are affected by nutrient access and utility [7]. According to Karabegović et al. [66] the use of *M. pulcherrima* in Prokupac grape must fermentation may also add to the content of polyphenols, anthocyanins and flavonoids and improve the color attributes of the resulting wines. This was explained by the low efficiency of *M. pulcherrima* to absorb anthocyanins in the cell wall.

Although iron scavenging has been established as the basic mechanism related to the antagonistic advantage of *M. pulcherrima* over other yeasts, additional mechanisms are also suspected to be involved in its interactions with competing species [38]. Experimental approaches studying, for instance, the effect of nutrient competition or cell contact on *M. pulcherrima*–*S. cerevisiae* interactions would add knowledge on the manner in which *M. pulcherrima* manages the presence of other wine yeasts.

## 4. Interactions between *Saccharomyces* Species

The growing concern about global warming has prompted further study into yeast interactions. The temperature increase affects the concentration of sugars in grapes, thus giving rise to products with higher ethanol content [67]. This, in combination with modern consumer demands for wines with less ethanol and more complex aromas has motivated researchers to look for new mixed-species starters that may respond to these challenges.

Fermentation at low temperatures is becoming a trend for the enrichment of the wine aroma [68]. Recent studies have been mining for interactions between *S. cerevisiae* and other *Saccharomyces* species (*Saccharomyces* non-*cerevisiae* yeasts, SNC) as well as their hybrids. Compared to non-*Saccharomyces* yeast species, which do not have such high competitive fitness and efficiency during fermentation, SNC yeasts may possess desirable characteristics in order to fulfill the requirements of the wine industry. SNC yeasts such as *S. kudriavzevii*, *S. paradoxus*, *S. uvarum*, *S. eubayanus* and their interspecific hybrids such as *S. cerevisiae* × *S. uvarum* and *S. cerevisiae* × *S. kudriavzevii* are considered to be good low-temperature fermenters and have shown great potential in producing wines with reduced ethanol and increased glycerol content [69]. Among SNC yeasts, *S. uvarum* has been found in wine fermentations of cold-climate regions, while the other species have been isolated from wild environments. On the contrary, various *Saccharomyces* interspecific hybrids have been found in wine fermentation environments of cool climates. For instance, while *S. kudriavzevii* is an oak bark isolate, its hybrids with *S. cerevisiae* and *S. uvarum* (i.e., *S. cerevisiae* × *S. kudriavzevii* and *S. cerevisiae* × *S. kudriavzevii* × *S. uvarum*) have been found to dominate in wine fermentations. In addition, these natural hybrids have a stronger competitive advantage compared to their parental species, since they often combine the beneficial adaptive traits of their parents (e.g., ethanol and cryotolerance or resistance to other stressors). Both SNC species and their hybrids are considered of relevance to winemaking due to their positive oenological properties [68,70,71,72,73,74,75,76]. Recent work has shown that *S. uvarum* was established and persisted in a Canadian winery, thus dominating over grape microbial populations during un-inoculated Chardonnay fermentations [77]. Such observations, which are also common for white wines across European regions with cool climates, may suggest a competitive advantage of *S. uvarum* and/or other cryotolerant SNC species under low temperatures. Corroborating this proposition, Alonso-del-Real et al. [25] found that the advantage in growth and fermentation performance of *S. cerevisiae* compared to four different SNC yeasts at high temperatures seems to subside as the temperature decreases. *S. cerevisiae* may co-exist with other SNCs, such as *S. eubayanus*, *S. kudriavzevii* and *S. paradoxus*, or even be displaced by *S. uvarum* in synthetic grape must at 12 °C. Interestingly, the co-inoculation of must with Sc and *S. kudriavzevii* at low temperatures increased the rate of sugar consumption compared to single-species fermentations, and proved to be beneficial for the composition (ethanol and glycerol content) of the final product. Evidently, temperature plays a determinant role in *Saccharomyces* interspecies competition. Consistent with this, Balsa-Canto et al. [78] constructed an ecological model with temperature-dependent parameters to describe the behavior of *S. cerevisiae* and *S. kudriavzevii* in mixed-culture fermentation systems. The work revealed that *S. cerevisiae* may accelerate its growth due to the presence of *S. kudriavzevii* and outperform its competitor at temperatures above 24 °C. However, at temperature ranges between 8 and 10 °C the two populations may coexist and benefit from each other. According to the authors, resource partitioning (nutrient availability, temperature, production of ethanol) allows for a fine tuning of this beneficial co-existence via inoculation of Sc 24 h after the addition of *S. kudriavzevii*.

Sequential inoculation may prove a promising strategy for mild temperature, mixed-*Saccharomyces* species fermentations. Indeed, recent evidence suggests that a sequential inoculation scheme combined with controlled air supply may boost the fitness of *S. kudriavzevii* in mixed fermentations with Sc [79]. The impact of aeration on *Saccharomyces* interspecies interactions apparently relates to Sc adaptation and better performance under anaerobic conditions. Alonso-del-Real et al. [31], after analyzing a combination of growth, metabolomics, and transcriptomic data, suggested that nutrient and particularly nitrogen uptake modulates the competition between *S. cerevisiae* and *S. kudriavzevii*. Interestingly, it was demonstrated that interactions between *Saccharomyces* species may be triggered by cell-to-cell contact. Using a realistic approach of Chardonnay must fermentations, Morgan et al. [28] found that the interactions between *S. cerevisiae* and *S. uvarum* were largely defined by temperature. A high temperature was shown to favor the dominance of Sc over *S. uvarum* and vice versa. Initial inoculum was also shown to be critical for the dominance of one species over the other. Ultimately, an inoculum consisting of an equal populations ratio led to species co-existence at 15 °C, and a product with a unique volatile profile was obtained.

Based on the above studies, an interplay between the inoculation strategy and the fermentation temperature is likely a crucial factor in the competition between Sc vs. SNC in mixed inocula. Apparently, the feasibility and the output of *Saccharomyces* mixed species fermentations are still unsettled. The properties of SNC species as well as of their hybrids should be further explored in relation to the benefit of their use in winemaking processes. In this context, comparative studies between mixed *Saccharomyces* species fermentations and fermentations using natural *Saccharomyces* interspecific hybrids may also be of additive value. A deeper insight into the physiological and metabolic responses of *Saccharomyces* species upon their interaction will open up the way to the optimal design of Sc–SNC co-fermentations. Such knowledge will also provide a better understanding of species’ ecological relationships and will allow for drawing conclusions regarding their establishment in natural habitats.

## 5. Insight into Interaction Mechanisms

Transcriptomics has become one of the most popular methods to gain information of the genes that play key roles in microbial responses to their environmental cues and under different stressors [80]. In this context, the assessment of microbial “mRNA status” in the presence of neighboring microorganisms is receiving research interest. Regarding yeast interactions under wine-making simulated conditions, transcriptomic studies have only recently started to show up and are still quite scarce. Thus far, these studies have mainly investigated the regulation of *S. cerevisiae* gene expression in response to the presence of other NS yeasts during alcoholic fermentation. It has been revealed that when in co-culture with other yeasts, a number of major metabolic pathways in *S. cerevisiae* may undergo a complete shift to cope with the biotic pressure induced by the presence of the competitor. For instance, such genes may be related to glucose or nitrogen uptake, acetic acid production and response to heat shock (Table 2). Due to the limited number of relevant studies, it is not easy to identify common transcriptional responses of *S. cerevisiae* dependent on competing species. A few studies exist solely with *T. delbrueckii* in co-culture with *S. cerevisiae*. Curiel et al. [81] showed that most Sc genes induced due to the presence of *T. delbrueckii* were under the control of nitrogen catabolite repression. Likewise, Ruiz et al. [54], investigating the effect of nitrogen availability on the transcriptional responses of Sc under co-inoculation with Td, demonstrated an upregulation of genes related to amino acid uptake and hexose transport. These authors also discussed the common mechanisms that Sc may activate to counteract biotic (e.g., Td presence) and abiotic (e.g., nitrogen starvation) stressors. Tronchoni et al. [82] found that *S. cerevisiae* could differentially express (mainly upregulate) genes of the glucose fermentation pathway, as well as *PAU* genes. However, all three studies reported the upregulation of *HSP12*, a gene encoding a heat shock protein, in response to *T. delbrueckii*. Previously, it was suggested that the upregulation of *HSP12* might occur as a stress response to surrounding cells [16,82]. *HSP12* has been shown to be involved in the improvement of sweetness in red dry wines [83]. This supports the notion that during mixed fermentations the secretion of molecules ascribed to yeast interactions might contribute to alterations of wine attributes and enhancement of the complexity perception. Interestingly, the changes in the transcriptome of Td due to the presence of Sc may point to different strategies employed by each species in order to efficiently defend themselves against competitors. For instance, following co-inoculation of Td–Sc in high sugar media, Tondini et al. [84] observed that Td activates genes related to the ESR (environmental stress response) pathway, while the *S. cerevisiae* response would rely more on the activation of specific branches of the HOG (high osmolarity glycerol) and CWI (cellwall integrity) pathways, as well as of glucose catabolite repression promoting growth and sugar uptake. Such studies are quite useful in the pursuit of hypotheses regarding the adaptation of each species to wine-related environments.

Kosel et al. [87], based on the assumption that cell–cell contact does not mediate competition between *S. cerevisiae* and *D. bruxellensis*, monitored the growth of the two microorganisms in mixed cultures but physically separated by a membrane, allowing exchange of metabolites but not the passage of yeast cells. The authors studied the differential regulation of *S. cerevisiae* genes in the presence of *D. bruxellensis* and showed changes in the transcription of genes belonging to the *PAU* gene family. Interestingly, due to the absence of cell contact it may be inferred that the regulation of these genes provoked by yeast competitive interactions does not require cell contact, and it is apparently based on molecule excretion and sensing.

In a recent study [85], it was shown that both *L. thermotolerans* and *S. cerevisiae* reshape their transcriptome in response to co-culture by mainly changing the expression of cellwall integrity-associated genes (e.g., expression of five *PAU* genes in *S. cerevisiae* and increased expression of endoglucanases in *L. thermotolerans*). *S. cerevisiae* upregulated the systems associated with iron and copper acquisition in contrast to *L. thermotolerans*, in which downregulation of the respective genes was observed. The regulation of iron and copper transport-associated Sc genes seems to be critical under competition for nutrients. As stated by Ruiz et al. [54], cell homeostasis and competitive capacity is dependent on the efficient uptake of these elements.

The quick response of *S. cerevisiae* regarding the activation of mechanisms which will allow for efficient resource utilization apparently render it a successful competitor under oenological conditions. It is indeed evidenced that even when the competing microorganism upregulates genes to respond to stress (e.g., oxidative or osmotic), this response seems delayed compared to that of *S. cerevisiae* [82]. In agreement with this, an elegant study of Alonso-del-Real et al. [31] suggested a faster transcriptome remodeling of *S. cerevisiae* compared to *S. kudriavzevii* in response to competition. These authors investigated the role of yeast genetic relatedness in the mechanisms of yeast competition using two different strains of *S. cerevisiae*, one of wine and one of oak origin, each of them in co-inoculated fermentation with *S. kudriavzevii*. Despite their genetic similarity, the oak *S. cerevisiae* strain was a poor competitor in comparison to the wine strain and similarly to *S. kudriavzevii* did not manage to alter its transcriptome with the speed and extensiveness that the wine *S. cerevisiae* did. Interestingly, *S. kudriavzevii* responded to the presence of both strains in the same manner regarding transcriptional changes.

Apparently, the various conditions and strains used in different studies influenced the type of yeast–yeast interactions and the mode of gene regulation. There is limited research at the moment to allow for more solid comparisons or meaningful correlations. Even though the picture of species interactions is still not clear, studies trying to understand yeast interactions at the strain level have started to emerge. Pérez-Torrado et al. [32] attempted to gain insight into *S. cerevisiae* intra-species interactions since such information could possibly explain the dominance of specific *S. cerevisiae* strains—among an initially mixed strain population—at the end of alcoholic fermentations. The authors proposed a mechanistic model in which the upregulation of the *SSU1* gene (related to sulfite resistance) in combination with the expression of several cell surface proteins (related to cell aggregation) may provide a competitive advantage to the dominating strains.

Peng et al. [86] investigated the response of *S. cerevisiae* to the presence of *L. thermotolerans* during alcoholic fermentation at the translational level. Both the cellular and extracellular proteome was analyzed with the use of MS-based quantitative proteomics, combined with tandem mass tag labeling. Having observed an upregulation of stress response and metabolism proteins (e.g., heat shock proteins and ergosterol biosynthesis proteins) of *S. cerevisiae* at the first stages of co-cultivation with *L. thermotolerans*, in contrast to an increase in protein synthesis and enzymatic activity after the death of *L. thermotolerans*, they concluded that the stress response mechanisms in *S. cerevisiae* begin to shut down only when the majority of the competing population is no longer viable. This allows *S. cerevisiae* to start working on the production of proteins to optimize its survival during stationary growth phase. Both Peng et al. [86] and Shekhawat et al. [85], analyzing proteomic and transcriptomic data, respectively, point towards a significant triggering of several *S. cerevisiae* biological processes such as cell wall remodeling in the presence of *L. thermotolerans*. However, the essential differences of the two studies regarding the experimental setup and the time points selected for the analyses do not allow for robust assumptions regarding a link between *S. cerevisiae* transcriptional and translational responses under competition with *L. thermotolerans*.

Taken together, the results of the available literature cannot yet fully assemble the puzzle of wine yeast interactions. Studies on Sc/NS mixed-culture fermentations are still limited, while relevant research on Sc/SNC or NS/NS is still at its infancy. At present, there is no sufficient information to allow for the association of specific responses or interaction types with certain species. Future work will allow for the observation of changes in patterns of transcripts and metabolites in response to the presence of closely or distantly related yeast species and strains.

## 6. Concluding Comments

Grape must is a treasure chest of various yeast species and strains, and wine is actually a product of their interactions. Going with the flow of modern lifestyle and market trends, winemaking embraces diversity and promotes the exploitation of this rich grape microbiota for the enhancement of the wine’s individual character. This approach does not rule out studies that are based on empirical design and practical observations regarding optimum yeast mixtures. However, in order to ultimately develop a rational strategy and successfully implement oenological practices, a deep understanding of the fundamental principles underlying the function of the wine ecosystem is required. Walking through the era of microbiomics, holistic approaches combining phenotypic data along with transcriptomics, proteomics and metabolomics will allow for meaningful correlations and interpretations of yeast interactions. Such knowledge is essential for the comprehension of yeast community structure and dynamics in the grape–wine system and necessary for the innovation of wine fermentations.

## Figures and Tables

**Table 2:** Overview of recent studies using gene or protein expression analysis in wine yeast interactions research.

Yeast Species ^1^	Fermentation Conditions ^2^ (Inoculation Modality—Medium—Other Evaluated Parameters)	Method Applied	Experimental Approach and Implementations	Remarks ^3^	
Sc–Td	SM—High sugar SGM—Fermentation stage	RNA-seq analysis of yeasts transcriptomes in single vs. mixed cultures	Use of chimeric Sc/Td transcriptome for high-quality reads alignment	Species-specific transcriptomic response to competition and high-sugar environment; *ESR* genes in Td and HOG pathway or glycerol catabolic pathways genes in Sc	[84]
Sc–Td	SM—HN or LN SGM—Fermentation stage	RNA-seq analysis of Sc transcriptome in single vs. mixed cultures	Multiple comparisons for identification of competition-specific transcriptional responses	Stronger induction of Sc genes under HN in response to competition	[54]
Sc–Td	SM—SGM—2 vs. 12–14h interaction	RNA-seq analysis of Sc transcriptome in single vs. mixed cultures	Use of chimeric Sc/Td genome for reads mapping	Species-common response to biotic stress via *HSP12* induction. Delayed transcriptomic Td response to co-culture compared to Sc	[82]
Sc–Lt	SM—SGM—Aerobic or anaerobic environment	RNA-seq and global analysis of each transcriptome in single cultures vs. total transcriptome of the mixed culture	Chemostat-simulating fermentation system for stable populations and growth medium composition	Cell-wall integrity genes in both species. Upregulation of iron and copper acquisition systems in Sc vs. downregulation in Lt	[85]
Sc–Lt	SM—SGM—Lt death stage (early vs. late) at microaerobic environment	Tandem mass tag-based proteomics of Sc in single vs. mixed cultures	Cell staining and flow cytometry for species seperation, extraction of proteins from two cellular sub-fractions	Death phase-dependent protein expression; SRPs regulation indicative for increased Sc enzymatic activity at EDP and relief from stress at LDP	[86]
Sc–Mp	SQ—NGM—Different timepoints during fermentation	Transcriptional analysis of Sc genes involved in acetic acid and glycerol pathways in single vs. mixed cultures	Use of primers specific for Sc genomic DNA	Time-dependent redirection of genes involved in the acetic acid and glycerol production pathways	[65]
Sc–Db	SM—SGM—Fermentation stage, microaerobic conditions, restriction of yeast contact	Microarray analysis of Sc transcriptome in single vs. mixed cultures	Double compartment membrane fermentors for species separation	Key role of *PAU* gene family in Sc–NS competition	[87]
Sc–Hu or Sc–Cs or Sc–Td	SM—SGM—Early stages of interaction in aerobic environment	RNA-seq analysis of Sc transcriptome in single vs. mixed cultures	Focus at early fermentation stages and use of aerobic regime	Regulation of: *NCR* genes in Td; *CATT* and *SCMP* genes in Cs; “response to stimulus” and “response to stress” genes in Hu	[81]
Sc–Sk	SM—SGM—Fermentation temperature and stage	RNA-seq analysis of yeast transcriptomes in single vs. mixed cultures	Pair-end and read length sequencing for effective seperation of sequences from genomes of high identity	Nutrient uptake and cell division genes regulation favored by yeast competition; weaker response of Sk at EEP	[31]
Sc–Sc (dominant vs. non-dominant strain)	SM—NGM—Growth state of the non-dominant strain	RNA-seq analysis of strains transcriptomes in single vs. mixed cultures	Fluorescent labeling of strains and discrimination by flow cytometer	Regulation of 330 genes in non-dominant vs. 32 genes in dominant strain; competitive advantage of dominant strain via overexpression of *SSU1* sulphite resistance gene	[32]

^1^ Sc, *Saccharomyces cerevisiae*; Td, *Torulaspora delbrueckii*; Lt, *Lachancea thermotolerans*; Mp, *Metschnikowia pulcherrima*; Db, *Dekkera bruxellensis*; Hu, *Hanseniaspora uvarum*; Cs, *Candida sake*; Sk, *Saccharomyces kudriavzevii*.^2^ SM, simultaneous yeast species inoculation; SQ, sequential inoculation of *Saccharomyces cerevisiae* after NS species; SGM, synthetic grape must; NGM, natural grape must; HN/LN, high/low nitrogen;.^3^ ESR, environmental stress response; HOG, high osmolarity glycerol; EDP, early death phase; LDP, late death phase; NCR, nitrogen catabolite repression; CATT, carboxylic acid transmembrane transport; SCMP, sulfur compound metabolic process; SRP, significantly regulated protein; EEP, early exponential phase.
